# Survival of Patients Treated with Antibiotics and Immunotherapy for Cancer: A Systematic Review and Meta-Analysis

**DOI:** 10.3390/jcm9051458

**Published:** 2020-05-13

**Authors:** Fausto Petrelli, Alessandro Iaculli, Diego Signorelli, Antonio Ghidini, Lorenzo Dottorini, Gianluca Perego, Michele Ghidini, Alberto Zaniboni, Stefania Gori, Alessandro Inno

**Affiliations:** 1 Oncology Unit, ASST Bergamo Ovest, 24047 Treviglio, Italy; 2 Oncology Unit, ASST Bergamo Est, 24068 Alzano Lombardo, Italy; aleiaculli@gmail.com (A.I.); ldottorini@gmail.com (L.D.); 3 Oncology Unit, Fondazione IRCCS Istituto Nazionale Tumori di Milano, 20133 Milano, Italy; diegosignorelli@yahoo.it; 4 Oncology Unit, Casa di cura Igea, 20129 Milano, Italy; antonioghidini@hotmail.com; 5 Pharmacy Unit, IRCCS San Raffaele Hospital, 20132 Milano, Italy; gianperec@gmail.com; 6 Oncology Unit, Fondazione IRCCS Ca’ Granda Ospedale Maggiore Policlinico, 20122 Milano, Italy; Michele.ghidini@policlinico.mi.it; 7 Oncology Unit, Fondazione Poliambulanza, 25124 Brescia, Italy; azaniboni@alice.it; 8 Oncology Unit, IRCCS Ospedale Sacro Cuore Don Calabria, Negrar, 37024 Verona, Italy; stefania.gori@sacrocuore.it (S.G.); alessandro.inno@gmail.com (A.I.)

**Keywords:** cancer, immune checkpoint inhibitors, survival, antibiotic, meta-analysis

## Abstract

Antibiotics (ABs) are common medications used for treating infections. In cancer patients treated with immune checkpoint inhibitors (ICIs), concomitant exposure to ABs may impair the efficacy of ICIs and lead to a poorer outcome compared to AB non-users. We report here the results of a meta-analysis evaluating the effects of ABs on the outcome of patients with solid tumours treated with ICIs. PubMed, the Cochrane Library and Embase were searched from inception until September 2019 for observational or prospective studies reporting the prognoses of adult patients with cancer treated with ICIs and with or without ABs. Overall survival (OS) was the primary endpoint, and progression-free survival (PFS) was the secondary endpoint. The effect size was reported as hazard ratios (HRs) with a 95% confidence interval (CI) and an HR > 1 associated with a worse outcome in ABs users compared to AB non-users. Fifteen publications were retrieved for a total of 2363 patients. In the main analysis (*n* = 15 studies reporting data), OS was reduced in patients exposed to ABs before or during treatment with ICIs (HR = 2.07, 95%CI 1.51–2.84; *p* < 0.01). Similarly, PFS was inferior in AB users in *n* = 13 studies with data available (HR = 1.53, 95%CI 1.22–1.93; *p* < 0.01). In cancer patients treated with ICIs, AB use significantly reduced OS and PFS. Short duration/course of ABs may be considered in clinical situations in which they are strictly needed.

## 1. Introduction

Cancer immunotherapy with immune checkpoint inhibitors (ICIs) has demonstrated efficacy among several tumour types [1]. However, a non-negligible percentage of patients do not derive any benefit from ICIs, and the research for predictive factors may help to refine patients’ selection and improve treatment efficacy.

Preclinical studies on murine models have demonstrated that gut microbiota may act as a key modulator of efficacy and toxicity of ICIs [2,3]. Thus, it has been supposed that response to ICIs in humans could be affected by conditions that alter the composition of gut microbiota, including dysbiosis, due to the administration of antibiotics (ABs). In fact, retrospective studies reported worse outcomes for patients treated with ICIs that received ABs as compared with those which did not receive ABs [4,5,6]. 

The present meta-analysis evaluated the association between AB use and outcomes in patients with solid tumours treated with ICIs. 

## 2. Experimental Section

### 2.1. Search Strategy and Inclusion Criteria

This meta-analysis was conducted following the Preferred Reporting Items for Systematic Reviews and Meta-Analyses (PRISMA) guidelines [7]. A systematic search was performed using Embase, PubMed, SCOPUS and Cochrane Library databases. The search was performed until September 2019 using the terms antibiotics AND (PD-1 or PD-L1 or “immune checkpoint inhibitors” or CTLA-4) AND survival. All identified articles were then independently assessed for inclusion and exclusion criteria by two investigators (Alessandro Inno and Fausto Petrelli).

The inclusion criteria used for articles selection were the following: (1) adult patients with solid tumours and treated with ICIs, (2) evaluation of survival (OS and/or PFS) according to intake of ABs (yes versus no), (3) a hazard ratio (HR) statistic accompanied by a 95% confidence interval (CI) from univariate or adjusted Cox multivariate analysis and (4) inclusion of adult patients. The exclusion criteria were the following: (1) phase I studies and (2) patients treated with ICIs and other (non-immunotherapy) drugs. When different papers published series involving overlapping patients or more extended follow-up, the most updated reports were included for quantitative assessment. Only studies involving human subjects and published in English were included. 

### 2.2. Data Extraction

Two investigators (Alessandro Inno and Fausto Petrelli) independently extracted data (author and year of publication, number of patients, type of study, treatment received, timing of AB therapy, median follow-up and type of analysis). The quality of the included studies was determined with the Newcastle–Ottawa Scale (NOS) [8].

### 2.3. Statistical Analysis

The primary aim of this meta-analysis was the effect of AB intake on outcome, reported as HR and its respective 95% CI. Overall survival was the primary endpoint, and PFS was the secondary endpoint. The HRs of any included study were pooled together to provide the overall effect size. I^2^ statistic was used to provide an estimation of the percentage of total variation across studies, owing to heterogeneity. Values greater than 50% meant that substantial heterogeneity existed. A random-effect model was used in cases of high heterogeneity; otherwise, in the case of I^2^ < 50%, a fixed-effect model was appropriated [9]. Publication bias was assessed through the generation of funnel plots for OS and analysed for asymmetry using both the Begg and Egger test. All *p* values were two-sided with significance set at *p* < 0.05. Statistical analyses were conducted with the Review Manager computer program, Version 5.3 (Copenhagen: The Nordic Cochrane Centre, The Cochrane Collaboration, Copenhagen, Denmark 2014).

## 3. Results

**Figure 1:**
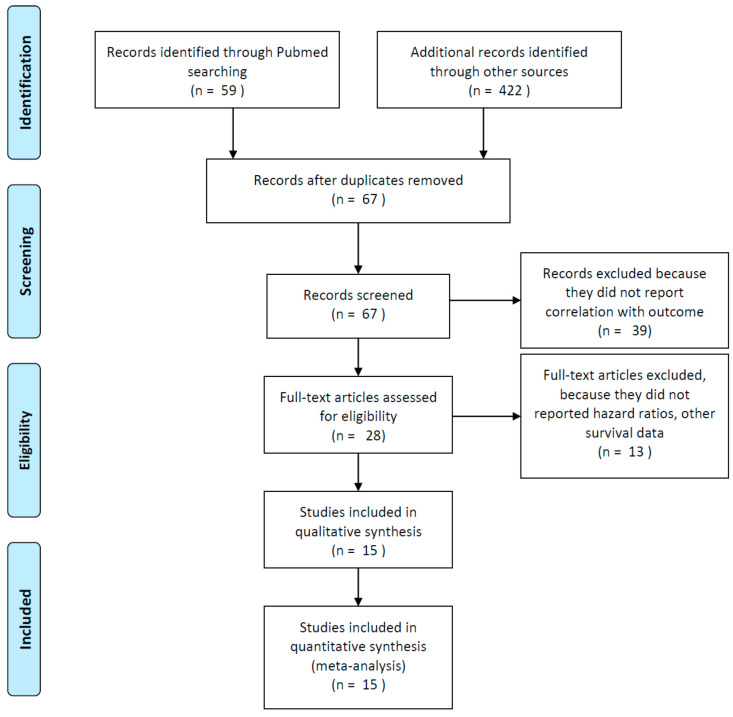
Flow diagram of included studies.

Among the publications retrieved using electronic search, 15 studies were eligible for quantitative analysis, for a total of 2363 patients [4,5,6,10,11,12,13,14,15,16,17,18,19,20,21] (Figure 1).

Baseline characteristics of the included studies and treatments received are reported in Table 1. Thirteen were retrospective series and two were prospective studies. Among the studies, 11, three and one included patients treated with ABs prior to and/or during ICIs and only prior to and only during ICIs, respectively. Median courses of ABs were rarely reported. In studies where median duration of antibiotics was reported, it was no longer than two weeks and no shorter than one week, respectively. 

**Figure 2:**
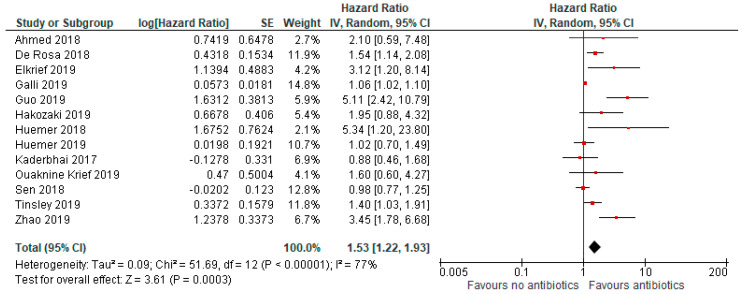
Forrest plot for progression-free survival in patients assuming antibiotics pre/during immunotherapy. IV, inverse variance; CI, confidence interval; SE, standard error.

The median age was 64 years. Antibiotics were given to 29% of patients. Progression-free survival was reduced in those who took antibiotics (HR = 1.53, 95% CI 1.22–1.93; *p* < 0.01; Figure 2). 

The analysis included nine studies, and due to high heterogeneity (I^2^ = 77%), a random effect model was adopted.

**Figure 3:**
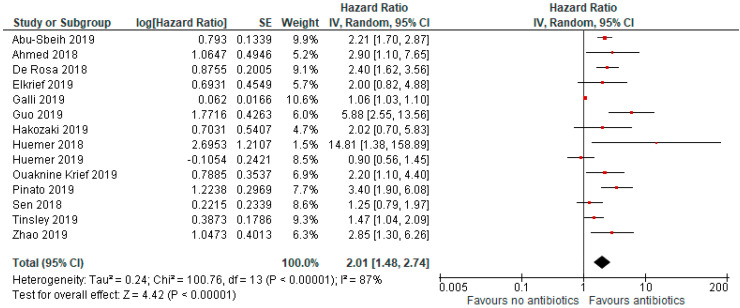
Forrest plot for overall survival in patients assuming antibiotics pre/during immunotherapy.

In the primary analysis, use of antibiotics was associated with an increased risk of death (HR = 2.07, 95% CI 1.51–2.84; *p* < 0.01; Figure 3). 

The analysis included 14 studies, and due to high heterogeneity (I^2^ = 87%), a random effect model was adopted. 

**Figure 4:**
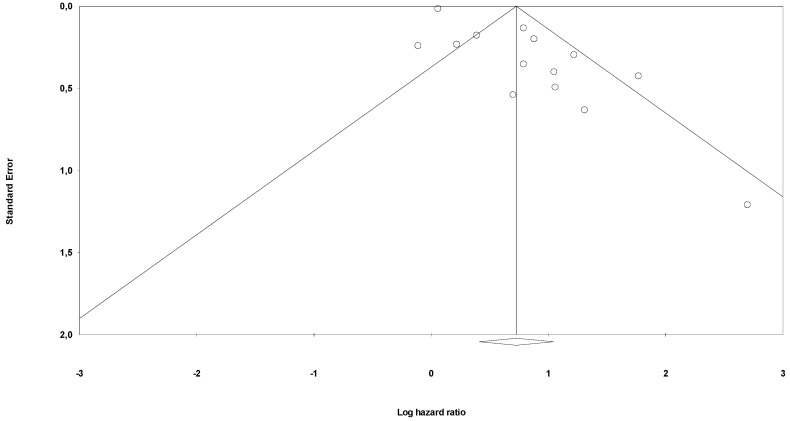
Funnel plot for publication bias (OS).

**Figure 5:**
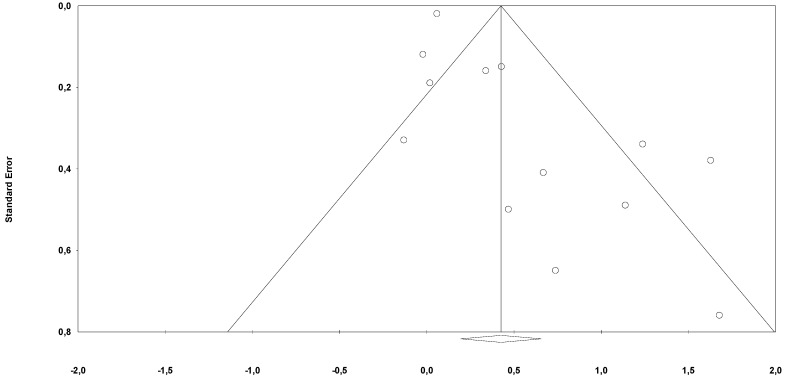
Funnel plot for publication bias (PFS)**.**

Risk of bias through Begg’s funnel plot was not significant for the OS and PFS analysis (Figure 4 and Figure 5). Conversely, Egger’s test showed evidence of bias (*p* < 0.01 for both analysis). After adjusting for missing studies through the trim and fill method, we found that the point estimate of the overall effect size remained significant only for OS analysis HR = 1.65 (95%CI, 1.25–2.17). After the one study removal procedure, we showed that after removing one study at a time the HRs ranged from 1.86 to 2.17, with the Guo et al. paper exerting the largest effect on OS.

Subgroup analysis was performed on the timing of antibiotics. Only one study included patients treated with ABs exclusively during ICIs (Galli et al.), and it did not report any reduced survival. Two other authors presented results of the effect of prior AB use with respect to start of ICIs (Derosa et al. and Elkrief et al.), and aggregated analysis of these two papers showed a similar effect size (HR = 2.33, 95%CI 1.33–3.34; *p* < 0.01). All other publications included a mixed group of patients given ABs before and/or during ICIs (survival data not split for timing), so a formal analysis of these studies was not presented but results were similar to main analysis (HR = 2.11, 95%CI 1.54–2.9; *p* < 0.01). Similarly, analysing the effect of administering AB classes or courses of ABs was not possible due to a lack of data. After excluding studies where analysis was not adjusted (univariate analysis), the effect on OS of AB use was more robust (HR = 2.33, 95%CI 1.61–2.37; *p* < 0.01). 

A meta-regression analysis was performed adjusting for median ABs duration, median follow up, disease sites and number of study patients (the only covariates with adequate number of data for analysis). No significant correlation was found to explain heterogeneity. 

## 4. Discussion

In the past years, it has been reported that changes in the gut microbiota of individuals with cancer who received antibiotics may reduce the outcome when they are treated with ICIs. We performed a systematic review and meta-analysis of observational evidence reporting the outcome of patients treated with ICIs for advanced cancers according to AB exposure, and we found that use of ABs reduces OS and PFS. 

In a seminal paper published in *Science* in 2018, Routy et al. [22] showed that AB consumption is associated with reduced response to the anti-PD-(L)1 blockade. Samples attained from patients with lung and kidney cancer showed that non-responding patients had low levels of the bacterium *Akkermansia muciniphila*. Oral bacterium supplementation in antibiotic-treated mice restored the response to immunotherapy. Gopalakrishnan et al. and Matson et al. [23,24] evaluated faecal samples from melanoma patients receiving anti-PD-(L)1 blockade and found that those who failed immunotherapy had an imbalance in commensal bacteria composition which was linked with impaired activity of immune cells. Other authors found that faecal *Bifidobacterium* was associated with the antitumor effects of ICIs3. Oral administration of *Bifidobacterium* alone also improved tumour control to the same magnitude as anti-PD-(L)1 therapy, and combination treatment nearly abolished tumour outgrowth. Increased dendritic cell function with a consensual enhanced cluster of differentiation 8 (CD8) + T cell priming/accumulation in the tumour microenvironment mediated the observed effect. Similarly, even the antitumor effect of Cytotoxic T-Lymphocyte Antigen 4 (CTLA-4) blockade seems to depend on distinct *Bacteroides* species, as found in mouse models by Vétizou et al. [25]. Lack of response was overcome by *B. fragilis* by immunization with *B. fragilis* polysaccharides or by adoptive transfer of *B. fragilis*-specific T cells; conversely, AB-treated mice did not respond to CTLA-4 blockade. 

In clinical settings, several authors reported a possible detrimental association between timing of/exposure to ABs and survival with ICIs. Particularly, Galli et al. [5] found that an elevated ratio between days of antibiotics and days of immunotherapy is more harmful than the use of ABs itself. In a similar study, Tinsley et al. [26] observed that a single course of ABs is associated with a better OS than that of multiple/prolonged courses of ABs. Although these observations are consistent with a possible detrimental effect of ABs, it cannot be excluded that AB use may identify a group of patients with poor prognoses due to concomitant severe infections or comorbidities, rather than ABs themselves affecting the outcome of patients treated with ICIs. 

Our meta-analysis has some limitations. First, this is a meta-analysis of retrospective series with heterogeneous populations and obvious diversity in tumour stages/types and patient characteristics. AB type and duration, as well as the indication of AB use, were only partially reported. Third, there is a potential bias linked to covariates used in con multivariate analysis, sample sizes, follow-up and clinical characteristic of populations included. Fourth, a direct association between AB use and the effect on microbiota and then on OS cannot be concluded because no concomitant evaluation of gut microbiota composition under antimicrobial influence was addressed. Finally, patients treated with anticancer therapy other than ICIs were not included. However, this pooled analysis of real-life experiences seems to confirm the hypothesis that AB-associated dysbiosis might be detrimental in patients treated with ICIs. A recently published paper by Huang and colleagues had the same goal of the present meta-analysis but with a less updated literature search, and about half of the included papers were congress abstract forms but they came to a similar conclusion [27]. 

## 5. Conclusions

An intact gut microbiota is needed to elicit the immune system and provide ICI benefits to cancer patients. Strategies to modulate the microbiome with the aim to improve ICI efficacy should be actively investigated.

## Figures and Tables

**Table 1:** Characteristics of included studies.

Author/ Year	Type of Study	N° Of Patients (Disease)	Treatment Received (%)	Median Age (Years)	Ab% /Timing	Median Duration (Weeks)/n° of AB Courses/pts	Med FUP (mos)	Type of Analysis	Covariates of MVA for OS	Quality (NOS Score)
Abu-Sbeih/ 2019	retrospective	826 (melanoma *n* = 347; hematologic *n* = 116; other *n* = 363)	anti-PD(L)1 (51.6), anti-CTLA4 (32), combo (16.5)	62	68.9 /before or after start (47.5%), both (52.5%)	NR/NR	NR	MVA	ICI type, Stage IV cancer, IMDC, anaerobic AB use	6
Ahmed/ 2018	retrospective	60 (NSCLC *n* = 34; other *n* = 26)	anti-PD1 (81.7), anti-PDL1 (5), ICI + CT (13.3)	59	28 /2w before and/or after start	1–2	NR	MVA	broad spectrum AB use, age	5
Derosa/ 2018	retrospective	360 (RCC *n* = 121, NSCLC *n* = 239)	RCC: anti-PD(L)1 (88), anti-PD(L)1 + anti-CTLA4 (8), anti-PD(L)1 + BEVA (4) NSCLC: anti-PD(L)1 (86), combo (14)	64	21.5 /1 mos before start	NR/NR	NR	MVA	RCC: ab 30–0 days/no AB IMDC risk, tumour burden NSCLC: ab 30–0 days/no AB, PS, clinical trial Y/N, prior regimens >/<3	5
Elkrief/ 2019	retrospective	59 (melanoma) *	NIVO/PEMBRO/IPI (100)	64.5	13.5° /1 month before	0.9/NR	NR	MVA	age, PS, gender, AB use, LDH, BRAF, line of tx, type of ICI	5
Galli/ 2019	retrospective	157 (NSCLC)	anti-PD(L)1 (95.6), anti-CTLA4 o combo (4.4)	66.7	17.2 /during ICI period	1/NR	28.6	MVA	high AB /immunotherapy exposure ratio through entire ICI period	8
Guo/ 2019	retrospective	49 (oesophageal)	anti-PD(L1) alone (61), combo (39)	56.7	43/2 mos before or 1 month after	1.42/NR	16.4	MVA	PS, treatment, n° of metastatic sites, NLR, antibiotic use	7
Hakozaki/ 2019	retrospective	90 (NSCLC)	NIVO (100)	68	14.4/1 month before start	>1 (84.6%)/	NR	MVA	driver mutations	6
Huemer/ 2018	retrospective	30 (NSCLC)	NIVO (83), PEMBRO (17)	NR	37/1 month before/after start	NR/NR	NR	MVA	sex, antibiotic use, ICI, EGFR/ALK mutations, line of tx, PDL1 status, immune-related adverse events	5
Huemer/ 2019	retrospective	142 (NSCLC)	NIVO, PEMBRO or ATEZO (100)	66	44/1 months before or after start	NR/NR	13.3	UVA	NR	7
Kaderbhai/ 2017	retrospective	74 (NSCLC)	NIVO (100)	67.5	20.3/3 months before or concurrent	1/NR	NR	UVA (PFS)	NR	5
Krief/ 2019	prospective cohort	72 (NSCLC)	NIVO (100)	68.8	42/2 months before or 1 month after start	1.35/1.7	16.6	MVA	AB use; KRAS mutations, gemmatimonadaceae on blood microbiome at baseline	7
Pinato/ 2019	prospective cohort	196 (NSCLC *n* = 118; melanoma *n* = 38; RCC *n* = 11; other *n* = 26)	anti-PD(L)1 (96)	68	29/1 month before or concurrent	NR/NR	NR	MVA	response to ICI, AB 0–30 days before ICI	6
Sen/ 2018	retrospective	172 (NSCLC *n* = 21; RCC *n* = 25; melanoma *n* = 16; sarcoma *n* = 16; other *n* = 94)	anti-CTLA4 (61), anti-PD1 (39)	60	33/during and up to 2 mos before	NR/NR	NR	UVA	NR	5
Tinsley/ 2019	retrospective	291 (melanoma *n* = 179, RCC *n* = 48, NSCLC *n* = 69)	NR	66	32/2w before up to 6w after start	NR/NR	NR	MVA	AB use, comorbidities, metastatic sites > 3, PS > 0	6
Zhao/ 2019	retrospective	109 (NSCLC)	anti-PD1 (52.3), anti-PD1 + CT (30.3), anti-PD1 + antiangiogenic (17.4)	62	18.3/1 mos before or after start	NR/NR	NR	MVA	AB use, PS	6

* only immunotherapy without chemotherapy; °: all patients; AB: antibiotic; mos: months; RCC: renal cell carcinoma; NSCLC: non-small-cell lung cancer; PD1: programmed death 1; PDL1: programmed death-ligand 1; ICI: immune checkpoint inhibitors; CT: chemotherapy; CTLA4: Cytotoxic T-lymphocyte antigen 4; BEVA: bevacizumab; NIVO: nivolumab; PEMBRO: pembrolizumab; IPI: ipilimumab; ATEZO: atezolizumab; MVA: multivariate analysis; UVA: univariate analysis; PFS: progression-free survival; IMDC: international metastatic RCC database consortium; PS ECOG: performance status; tx: therapy; NLR: neutrophil to lymphocyte ratio; NR: not reported; AB: antibiotics; combo: combination of two immune checkpoint inhibitors.

## References

[1] Ribas A., Wolchok J.D. (2018). Cancer immunotherapy using checkpoint blockade. Science.

[2] Gori S., Inno A., Belluomini L., Bocus P., Bisoffi Z., Russo A., Arcaro G. (2019). Gut microbiota and cancer: How gut microbiota modulates activity, efficacy and toxicity of antitumoral therapy. Crit. Rev. Oncol..

[3] Sivan A., Corrales L., Hubert N., Williams J.B., Aquino-Michaels K., Earley Z.M., Benyamin F.W., Lei Y.M., Jabri B., Alegre M.-L. (2015). Commensal Bifidobacterium promotes antitumor immunity and facilitates anti-PD-L1 efficacy. Science.

[4] DeRosa L., Hellmann M., Spaziano M., Halpenny D., Fidelle M., Rizvi H., Long N., Plodkowski A., Arbour K., Chaft J. (2018). Negative association of antibiotics on clinical activity of immune checkpoint inhibitors in patients with advanced renal cell and non-small-cell lung cancer. Ann. Oncol..

[5] Galli G., Triulzi T., Proto C., Signorelli D., Imbimbo M., Poggi M., Fucà G., Ganzinelli M., Vitali M., Palmieri D. (2019). Association between antibiotic-immunotherapy exposure ratio and outcome in metastatic non small cell lung cancer. Lung Cancer.

[6] Tinsley N., Zhou C., Tan G., Rack S., Lorigan P.C., Blackhall F., Krebs M., Carter L., Thistlethwaite F., Graham D. (2019). Cumulative Antibiotic Use Significantly Decreases Efficacy of Checkpoint Inhibitors in Patients with Advanced Cancer. Oncologist.

[7] Moher D., Liberati A., Tetzlaff J., Altman D.G., Group P. (2009). Preferred reporting items for systematic reviews and meta-analyses: The PRISMA statement. BMJ.

[8] Wells G., Shea B., O’Connell D. The Newcastle-Ottawa Scale (NOS) for Assessing the Quality if Nonrandomizes Studies in Meta-Analyses. http://www.ohri.ca/programs/clinical_epidemiology/oxford.asp.

[9] Higgins J.P.T., Thompson S.G., Deeks J.J., Altman U.G. (2003). Measuring inconsistency in meta-analyses. BMJ.

[10] Abu-Sbeih H., Herrera L.N., Tang T., Altan M., Chaftari A.-M., Okhuysen P.C., Jenq R.R., Wang Y. (2019). Impact of antibiotic therapy on the development and response to treatment of immune checkpoint inhibitor-mediated diarrhea and colitis. J. Immunother. Cancer.

[11] Ahmed J., Kumar A., Parikh K., Anwar A., Knoll B.M., Puccio C., Chun H., Fanucchi M., Lim S.H. (2018). Use of broad-spectrum antibiotics impacts outcome in patients treated with immune checkpoint inhibitors. OncoImmunology.

[12] Elkrief A., El Raichani L., Richard C., Messaoudene M., Belkaid W., Malo J., Belanger K., Miller W., Jamal R., Letarte N. (2019). Antibiotics are associated with decreased progression-free survival of advanced melanoma patients treated with immune checkpoint inhibitors. OncoImmunology.

[13] Guo J.-C., Lin C.-C., Lin C.-Y., Hsieh M.-S., Kuo H.-Y., Lien M.-Y., Shao Y.-Y., Huang T.-C., Hsu C.-H. (2019). Neutrophil-to-lymphocyte Ratio and Use of Antibiotics Associated with Prognosis in Esophageal Squamous Cell Carcinoma Patients Receiving Immune Checkpoint Inhibitors. Anticancer Res..

[14] Hakozaki T., Okuma Y., Omori M., Hosomi Y. (2019). Impact of prior antibiotic use on the efficacy of nivolumab for non-small cell lung cancer. Oncol. Lett..

[15] Huemer F., Lang D., Westphal T., Gampenrieder S.P., Hutarew G., Weiss L., Hackl H., Lamprecht B., Rinnerthaler G., Greil R. (2019). Baseline Absolute Lymphocyte Count and ECOG Performance Score Are Associated with Survival in Advanced Non-Small Cell Lung Cancer Undergoing PD-1/PD-L1 Blockade. J. Clin. Med..

[16] Huemer F., Rinnerthaler G., Westphal T., Hackl H., Hutarew G., Gampenrieder S.P., Weiss L., Greil R. (2018). Impact of antibiotic treatment on immune-checkpoint blockade efficacy in advanced non-squamous non-small cell lung cancer. Oncotarget.

[17] Kaderbhai C., Richard C., Fumet J.D., Aarnink A., Foucher P., Coudert B., Favier L., Lagrange A., Limagne E., Boidot R. (2017). Antibiotic Use Does Not Appear to Influence Response to Nivolumab. Anticancer Res..

[18] Krief J.O., De Tauriers P.H., Duménil C., Neveux N., Dumoulin J., Giraud V., Labrune S., Tisserand J., Julie C., Emile J.-F. (2019). Role of antibiotic use, plasma citrulline and blood microbiome in advanced non-small cell lung cancer patients treated with nivolumab. J. Immunother. Cancer.

[19] Pinato D.J., Howlett S., Ottaviani D., Urus H., Patel A., Mineo T., Brock C., Power D., Hatcher O., Falconer A. (2019). Association of Prior Antibiotic Treatment with Survival and Response to Immune Checkpoint Inhibitor Therapy in Patients With Cancer. JAMA Oncol..

[20] Sen S., Pestana R.C., Hess K., Viola G., Subbiah V. (2018). Impact of antibiotic use on survival in patients with advanced cancers treated on immune checkpoint inhibitor phase I clinical trials. Ann. Oncol..

[21] Zhao S., Gao G., Li W., Li X., Zhao C., Jiang T., Jia Y., He Y., Li A., Su C. (2019). Antibiotics are associated with attenuated efficacy of anti-PD-1/PD-L1 therapies in Chinese patients with advanced non-small cell lung cancer. Lung Cancer.

[22] Routy B., Le Chatelier E., DeRosa L., Duong C.P., Alou M.T., Daillère R., Fluckiger A., Messaoudene M., Rauber C., Roberti M.P. (2017). Gut microbiome influences efficacy of PD-1–based immunotherapy against epithelial tumors. Science.

[23] Gopalakrishnan V., Spencer C.N., Nezi L., Reuben A., Andrews M.C., Karpinets T.V., Prieto P.A., Vicente D., Hoffman K., Wei S.C. (2017). Gut microbiome modulates response to anti–PD-1 immunotherapy in melanoma patients. Science.

[24] Matson V., Fessler J., Bao R., Chongsuwat T., Zha Y., Alegre M.-L., Luke J.J., Gajewski T.F. (2018). The commensal microbiome is associated with anti–PD-1 efficacy in metastatic melanoma patients. Science.

[25] Vétizou M., Pitt J.M., Daillère R., Lepage P., Waldschmitt N., Flament C., Rusakiewicz S., Routy B., Roberti M.P., Duong C.P. (2015). Anticancer immunotherapy by CTLA-4 blockade relies on the gut microbiota. Science.

[26] Tinsley N., Zhou C., Villa S., Tan G., Lorigan P.C., Blackhall F.H., Elliott T., Krebs M.G., Carter L., Thistlethwaite F. (2018). Cumulative antibiotic use and efficacy of immune checkpoint inhibitors in patients with advanced cancer. J. Clin. Oncol..

[27] Huang X.-Z., Gao P., Song Y.-X., Xu Y., Sun J.-X., Chen X.-W., Zhao J.-H., Wang Z.-N. (2019). Antibiotic use and the efficacy of immune checkpoint inhibitors in cancer patients: A pooled analysis of 2740 cancer patients. OncoImmunology.

